# Control Theory and Cancer Chemotherapy: How They Interact

**DOI:** 10.3389/fbioe.2020.621269

**Published:** 2021-01-14

**Authors:** Paola Lecca

**Affiliations:** Faculty of Computer Science, Free University of Bozen-Bolzano, Bolzano, Italy

**Keywords:** optimal control theory, cancer chemotherapy, pharmacokinetics/pharmacodynamics models, cell-phase specific chemotherapics, parameter sensitivity analysis and controls

## Abstract

Control theory arises in most modern real-life applications, not least in biological and medical applications. In particular, in biological and medical contexts, the role of control theory began to take shape in the early 1980s when the first works appeared on the application of control theory in models of pharmacokinetics and pharmacodynamics for antitumor therapies. Forty years after those first works, the theory of control continues to be considered a mathematical analysis tool of extreme importance and usefulness, but the challenges it must overcome in order to manage the complexity of biological processes are in fact not yet overcome. In this article, we introduce the reader to the basic ideas of control theory, its aims and its mathematical formalization, and we review its use in cell phase-specific models for cancer chemotherapy. We discuss strengths and limitations of the control theory approach to the analysis pharmacokinetics and pharmacodynamics models, and we will see that most of them are strongly related to data availability and mathematical form of the model. We propose some future research directions that could prove useful in overcoming the these limitations and we indicate the crucial steps preliminary to a useful and informative application of control theory to cancer chemotherapy modeling.

## 1. Introduction

Control theory is concerned with establishing whether the evolution of a system is controllable, i.e., whether the evolution can be influenced by some external agent, called *control*. *Optimal* control theory deals with finding a control for the system over a period of time such that a performance criterion is optimized. Control theory (and its dual, i.e., observability theory) was applied to the analysis of deterministic models of biological and biochemical processes (Lecca and Re, 2019; Thomas et al., 2019; Wu et al., 2019), and in particular to models of cancer chemotherapy. Mathematically, cancer chemotherapy can be formulated as an *optimal* control problem. This approach is more relevant than ever today, as testified by recent works (Schättler and Ledzewicz, 2015a,b; Wang and Schättler, 2016; Angaroni et al., 2020; Jarrett et al., 2020; Sweilam et al., 2020). In literature, applications of optimal control theory to mathematical models of cancer biology and role of chemotherapy began to appear in the 1980s and have appeared with regularity in the following years to the present day. The reader can refer to some of these works, such as for example (Swan, 1980, 1988, 1990; Martin, 1992; Swierniak et al., 1996; Kimmel and Swierniak, 2005; Pillis et al., 2008; Collins et al., 2010; Batmani and Khaloozadeh, 2011; Ledzewicz et al., 2013; Ghaffari et al., 2014; Michor and Beal, 2015; Wang and Schättler, 2016; Carrère, 2017; Irurzun-Arana et al., 2018). Indeed, a tumor undergoing pharmacological treatment can be viewed as a control system with the state of the system, given by the number of cancer cells at time *t*, *N*(*t*), and the control input at time *t*, *u*(*t*). Usually in these models *u*(*t*) is the drug dosage or the effect the drugs have on normal and cancer cells. Since chemotherapeutic agents affect both healthy cells and cancer cells, the objective of the control problem is to minimize the number of cancer cells keeping the toxicity to the normal tissue at a safe level. The greater usefulness of the optimal control model of cancer chemotherapy lies in the capacity of determining drug schedules that most effectively reduce the size of a tumor after a fixed period of treatment has elapsed (Martin, 1992; Ledzewicz and Schaettler, 2004).

Mathematical models for cancer chemotherapy are necessarily cell-cycle specific, because most drugs are active in a specific phase of the cell-cycle as reported in Ledzewicz and Schaettler (2004), Schättler and Ledzewicz (2015a). The starting point of cell cycle is a growth phase G1 after which the cell enters a phase S where DNA synthesis occurs. Then a second growth phase G2 takes place in which the cell prepares for mitosis (phase M) during which cell division occurs. Each of the two daughter cells can either re-enter phase G1 or for some time may simply lie dormant (phase G0) until re-entering G1, thus re-starting the entire process. The molecules of the anticancer drug treatments can be cell-killing agents, but also blocking and recruitment agents. Blocking agents slow down the transitions of the cells through the cell cycle, i.e., they slow down the tumor growth; recruiting agents make cancer cells leave the dormant stage G0 where they are not susceptible to any chemotherapy (Ledzewicz and Schaettler, 2004; Schättler and Ledzewicz, 2015a,b). Usually, in these models the phases of the cell cycle are clustered into compartments with the state representing the average number of cells in each compartment and the control representing the dosages or effects of the various drugs. The number of compartments depends on the number and types of chemotherapeutic agents considered, since as we said a chemotherapeutic agents may acts on one or more phases. The in- and out-flows between the compartments in the presence of the control, determine the system dynamics. We consider extremely important as a future line of progress in this field, the construction of a pharmacokinetic/pharmacodynamic model that is less abstract than those usually used and which mainly considers two variables, such as drug dosage and the number of cells exposed to them. In particular, we are thinking of a model that describes the interactions at the molecular level of the drug with the biological networks that inside the cell are responsible for its processing and with the molecular targets for which it is designed.

## 2. The Optimal Control Problem

The formulation of an optimal control problem consists of (i) a mathematical description of the problem to be controlled; (ii) a statement of the physical constraints, and (iii) the specification of a performance criterion (Kirk, 1970). We restrict our focus to models of ordinary differential equations. Let *x*_1_(*t*), *x*_2_(*t*), …, *x*_*n*_(*t*) be the state variables of the system at time *t*, and let *u*_1_(*t*), *u*_2_(*t*), …, *n*_*m*_(*t*) the inputs to the systems at time *t*. The system can be described by *n* differential equations

$$\begin{array}{l} \frac{dx_{1}}{dt}=f_{1}\left(x_{1}\left(t\right),x_{2}\left(t\right),\ldots ,x_{n}\left(t\right),u_{1}\left(t\right),u_{2}\left(t\right),\ldots ,u_{m}\left(t\right)\right)\\ \frac{dx_{2}}{dt}=f_{2}\left(x_{1}\left(t\right),x_{2}\left(t\right),\ldots ,x_{n}\left(t\right),u_{1}\left(t\right),u_{2}\left(t\right),\ldots ,u_{m}\left(t\right)\right)\\ \;\vdots \\ \frac{dx_{n}}{dt}=f_{n}\left(x_{1}\left(t\right),x_{2}\left(t\right),\ldots ,x_{n}\left(t\right),u_{1}\left(t\right),u_{2}\left(t\right),\ldots ,u_{m}\left(t\right)\right) \end{array}$$

where *f*_*i*_:ℝ → ℝ (*i* = 1, 2, …, *n*). In matrix form, we write

(1) $$\begin{array}{r} \frac{d\text{u}}{dt}=\text{f}\left(\text{x}\left(t\right),\text{u}\left(t\right),t\right) \end{array}$$

where

$$\begin{array}{ll} \text{x}\left(t\right)=\left[\begin{array}{c} x_{1}\left(t\right)\\ x_{2}\left(t\right)\\ \vdots \\ x_{n}\left(t\right) \end{array}\right]\;\text{u}\left(t\right)=\left[\begin{array}{c} u_{1}\left(t\right)\\ u_{2}\left(t\right)\\ \vdots \\ u_{m}\left(t\right) \end{array}\right] & \;\text{f}\left(t\right)=\left[\begin{array}{c} f_{1}\left(\text{x}\left(t\right),\text{u}\left(t\right),t\right)\\ f_{2}\left(\text{x}\left(t\right),\text{u}\left(t\right),t\right)\\ \vdots \\ f_{n}\left(\text{x}\left(t\right),\text{u}\left(t\right),t\right) \end{array}\right]. \end{array}$$

The explicit presence of *t* in the right-hand side of Equation (1) indicates that the system is *time-variant*. For a time-invariant system instead we will write

(2) $$\begin{array}{r} \frac{d\text{u}}{dt}=\text{f}\left(\text{x}\left(t\right),\text{u}\left(t\right)\right). \end{array}$$

If a system is time-varying and linear its state equations are

(3) $$\begin{array}{r} \frac{d\text{x}}{dt}=\text{A}\left(t\right)\text{x}\left(t\right)+\text{B}\left(t\right)\text{u}\left(t\right) \end{array}$$

where **A**(*t*) and **B**(*t*) are, respectively, *n* × *n* and *n* × *m* matrices with time-dependent elements. State equations for linear time-invariant systems are instead of the form

(4) $$\begin{array}{r} \frac{d\text{x}}{dt}=\text{A}\text{x}\left(t\right)+\text{B}\text{u}\left(t\right) \end{array}$$

where **A** and **B** are time-independent matrices.

A *history* of the control input **u**(*t*) in the interval [*t*_0_, *t*_*f*_] is called a *control history*. A *history* of the state variables **x**(*t*) in the interval [*t*_0_, *t*_*f*_] is called a *state trajectory*. The physical constraints are defined by the boundary conditions, i.e., the values of the state variables and the control input at the initial time *t*_0_ and *t*_*f*_. A state trajectory that satisfies the state variable constraints during the entire time interval [*t*_0_, *t*_*f*_] is called an *admissible trajectory*. A control history that satisfies the control constraints during the entire time interval [*t*_0_, *t*_*f*_] is called an *admissible control*. The admissibility is a useful property because it reduces the number of values that can be assumed by the state variable and the controls. Thanks to this property, instead of investigating all controls and trajectories histories to identify which is the optimal ones, we can analyse only those controls and trajectories that are admissible (Kirk, 1970).

The state variables that can be measured are called *outputs*. Let *y*_1_(*t*), *y*_2_(*t*), …, *y*_*p*_(*t*) denote the outputs, then

(5) $$\begin{array}{r} \text{y}=\text{g}\left(\text{x}\left(t\right),\text{u}\left(t\right),t\right). \end{array}$$

If the outputs are related to the states and controls by a time-varying relationship then

(6) $$\begin{array}{r} \text{y}=\text{C}\left(t\right)\text{x}\left(t\right)+\text{D}\left(t\right)\text{u}\left(t\right) \end{array}$$

where **C** and **D** are *p* × *n* and *p* × *m* time-dependent matrices. If the systems is time-invariant then **C** and **D** are time-independent matrices.

Once the model and the physical constraints are specified, last step in the formulation of the optimal control problem is the definition of a performance measure useful to quantitatively assess the performance of the system. An *optimal control* is defined as one that minimizes (or maximizes) the performance measure. The optimal control problem consists in finding an admissible control **u**_*a*_ which causes the system described by Equation (1) to follow an admissible trajectory **x**_*a*_ that minimizes the performance measure

(7) $$\begin{array}{r} J=h_{1}\left(\text{x}\left(t_{f}\right),t_{f}\right)+\int_{t_{0}}^{t_{f}}h_{2}\left(\text{x}\left(t\right),\text{u}\left(t\right),t\right)dt \end{array}$$

where *h*_1_ and *h*_2_ are scalar functions. Starting from the initial state **x**(*t*_0_) = **x**_0_ and applying a control **u**(*t*) for *t* ∈ [*t*_0_, *t*_*f*_] causes the systems to follow some trajectory. The performance measure *J* assign a real number to each trajectory. We refer the reader to Moore (2018), Lopes et al. (2009), Sargent (2000), and Alekseev et al. (1987) for a review about necessary optimality conditions.

Two important notes are duty here: (i) before attempting to determine the controls we have to make sure that the system is controllable, since we may not know in advance if a system is controllable and if an optimal control exists, and (ii) even if an optimal control exists, it may not be unique. In the first case we can resort to controllability theorems. In the second case, non-unique optimal controls may complicate the computational procedures, but they give the possibility of choosing among different control configurations (Kirk, 1970).

The general solution of Equation (4) is

(8) $$x(t)=e^{At}[x(t_{0})+\int_{t_{0}}^{t}e^{-A\tau }Bu(\tau )d\tau ].$$

Kalman (1960) has shown that a linear time-invariant system is controllable if and only if the *n* × *nm* matrix

(9) $$E\equiv [B|AB|A^{2}B|\cdots |A^{n-1}B]$$

has rank *n*. This condition guarantees that there are a finite time *t*_1_ ≥ *t*_0_, and a control **u**(*t*) (*t* ∈ [*t*_0_, *t*_*f*_]) which moves the state **x**_0_ = **x**(*t*_0_)) to time *t*_1_ (Kirk, 1970). In the following section, we specialize these concepts to a general dynamic model of cell-cycle specific antitumor treatments.

## 3. Cell-Cycle Specific Dynamic Model

We consider a multi-compartment and multi-drug model, that according to Ledzewicz and Schättler (Schättler and Ledzewicz, 2015a) is described by a bilinear system of the form

(10) $$\frac{dN}{dt}=N\left(A+\sum_{i=1}^{m}u_{i}B_{i}\right),\;N_{0}=N(0)$$

where *N* = *N*(*t*) is the number of cancer cell at time *t*, *m* is the number of drugs, *A* and *B* are *n* × *n* matrices, *n* is the number of compartments, and *u*_*i*_ represent the dosages of various drugs. The drugs performs a cytostatic action which kills both cancer and healthy cells.

A model for the objective function is given by the formalization of the aim therapeutic treatment, which is to kill the cancer cells while keeping the toxicity to the health cells acceptable. In literature there are many non-equivalent ways of modeling the objective function (Swan, 1990; Swierniak et al., 1996; Schättler and Ledzewicz, 2015b; Wang and Schättler, 2016; Angaroni et al., 2020). A model that is largely adopted was proposed by Świerniak (1995), Świerniak et al. (2016) with the objective to minimize the number of cancer cells at the end of a fixed therapy interval. In the context of this modeling, Ledzewiczs and Scättler (Schättler and Ledzewicz, 2015b) proposed a linear L1-type objective function of the form

(11) $$\begin{array}{r} J=pN\left(T\right)+\int_{0}^{T}\left(qN\left(t\right)+bu\left(t\right)\right)dt \end{array}$$

where *p* = (*p*_1_, *p*_2_, …, *p*_*n*_), *q* = (*q*_1_, *q*_2_, …, *q*_*m*_), and *b* = (*b*_1_, *b*_2_, …, *b*_*m*_). are non-zero row vectors of non-negative coefficients. The components of *b* corresponding to killing chemotherapeutic agents are positive. The term *pN*(*T*) is a weighted average of the total number of cancer cell at the end of a fixed therapy interval [0, *T*]. The term *qN*(*t*) prevents that the number of cancer cells rise to unacceptably high values at intermediate times. In this scheme, the toxicity of the drugs is only modeled indirectly through the term *bu*(*t*), i.e., the adverse effect on the healthy cells are represented only indirectly by minimizing the drug dosage *u*(*t*) in the objective function.

Usually the pharmacokinetics equation for the body/plasma drug concentration *c*(*t*) if a first-order differential equation as the following

(12) $$\begin{array}{r} \frac{dc}{dt}=-\left(k_{1}+k_{2}u\left(t\right)\right)c\left(t\right)+hu\left(t\right),\;c\left(0\right)=0 \end{array}$$

where *k*_1_ and *h* are two positive constants, but *k*_2_ is arbitrary.

Finally, the equations of the dynamics is

$$\begin{array}{l} \frac{dN}{dt}=\left(A+cB\right)N,\;N\left(0\right)=N_{0}\\ \frac{dc}{dt}=-[k_{1}+k_{2}u\left(t\right)]c\left(t\right)+hu\left(t\right),\;c\left(0\right)=0. \end{array}$$

This model is still incomplete, and thus unrealistic, since it lacks of the pharmacodynamics that takes into account the effect the drug concentration *c*(*t*) has on the cancer cells.

Generally, the effect can be modeled by a function

s:[0,∞)→[0,1].

Different models arise depending on the choice of the function *s*. Two commonly used models in practice are the so-called *E*_max_ model of the form

(13) $$\begin{array}{r} s_{1}\left(c\right)=E_{0}+\frac{E_{\text{max}}c}{EC_{50}+c} \end{array}$$

and the sigmoidal function

(14) $$\begin{array}{r} s_{2}\left(c\right)=E_{0}+\frac{E_{\text{max}}c^{k}}{EC_{50}^{k}+c^{k}} \end{array}$$

where *E*_0_ is the baseline of the drug efficacy, *E*_max_ if the maximum efficacy, *EC*_50_ is the potency and *k* is a positive integer greater than 1 (Macdougall, 2006; Choe and Lee, 2017). In the *E*_max_ model it is assumed that the drug becomes almost immediately effective, but then saturates at high concentrations. In the sigmoidal models the effectiveness at both lower and higher concentrations are more accurately approximated.

Ultimately, we obtain the following equations for dynamics

(15) $$\frac{dN}{dt}=[A+s(c)B]N,\;N(0)=N_{0}$$

(16) $$\frac{dc}{dt}=-[k_{1}+k_{2}u(t)]c(t)+hu(t),\;c(0)=0$$

and the objective function *J* remains the same as in Equation (11).

## 4. Model Simulation

**Figure 1:**
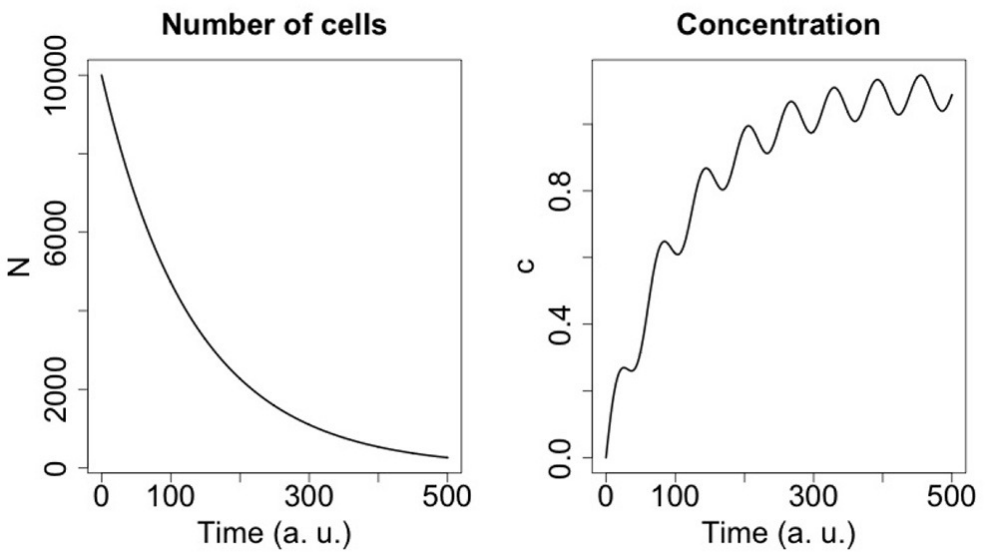
Numerical simulation of the system defined by Equations (15) and (16) with *E*_0_ = 0, *E*_max_ = 100, *k*_1_ = 0.005, *k*_2_ = 0.0004, *EC*_50_ = 15, *h* = 0.001, *k* = 1, *A* = 0.001, *B* = 0.001. As shown later, numerical simulations for times greater than 500 time units, shows that after the first 500 time units the oscillatory behavior disappears to give way to a plateau.

We simulated the model given in Equations (15)–(16) considering one compartment only (*A* and *B* are 1 × 1 matrices, i.e., scalar variables) and by setting the following values of the parameters expressed in arbitrary units: *E*_0_ = 0, *E*_max_ = 100, *k*_1_ = 0.005, *k*_2_ = 0.0004, *EC*_50_ = 15, *h* = 0.001, *k* = 1, *A* = 0.001, *B* = 0.001, and a time periodic function for the drug dosage *u*(*t*) = 10 cos(0.1*t*) + 10, to simulate a periodic administration of the drug. The values of *E*_max_, and *EC*_50_ have been taken as in Felmlee et al. (2012). The parameters *q*, *b* and *p* in the Equation (11) for *J* have been set equal to 1. The R code implementing the simulation of the models and the parameter sensitivity analysis is reported in Tables 1 and 2, respectively for the sake of results' reproducibility. The numerical solution of the equation systems if showed in Figure 1. The concentration curve shows synchronous fluctuations to the boulders and the minima of the *u*(*t*) curve and after a certain time interval it assumes a constant trend as long as the drug continues to be administered. In this model, if you stop administering the drug, the concentration curve assumes a decreasing trend after a certain time interval.

**Table 1:** R script implementing the model described by the Equations (15)–(16).

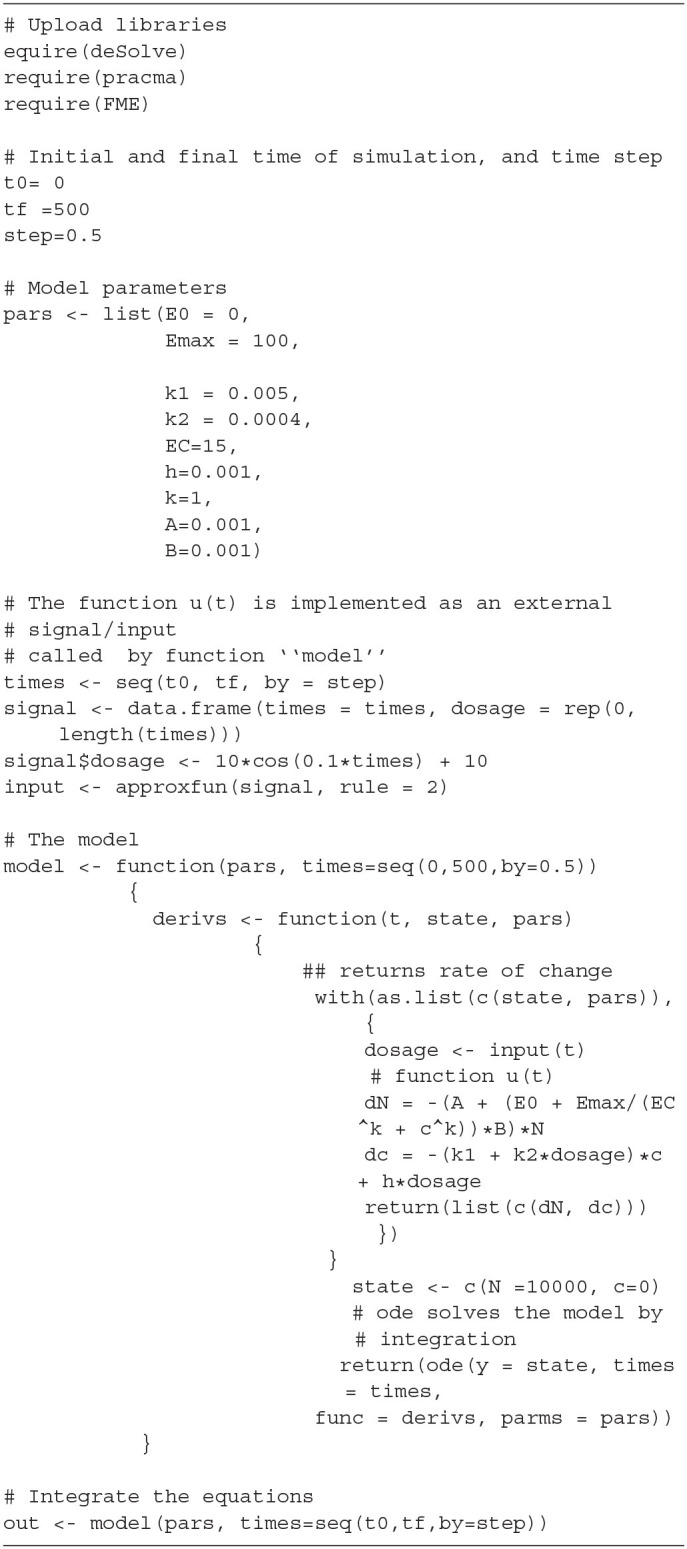

**Table 2:** R functions implementing the sensitivity analysis of the model of Equations (15)–(16).

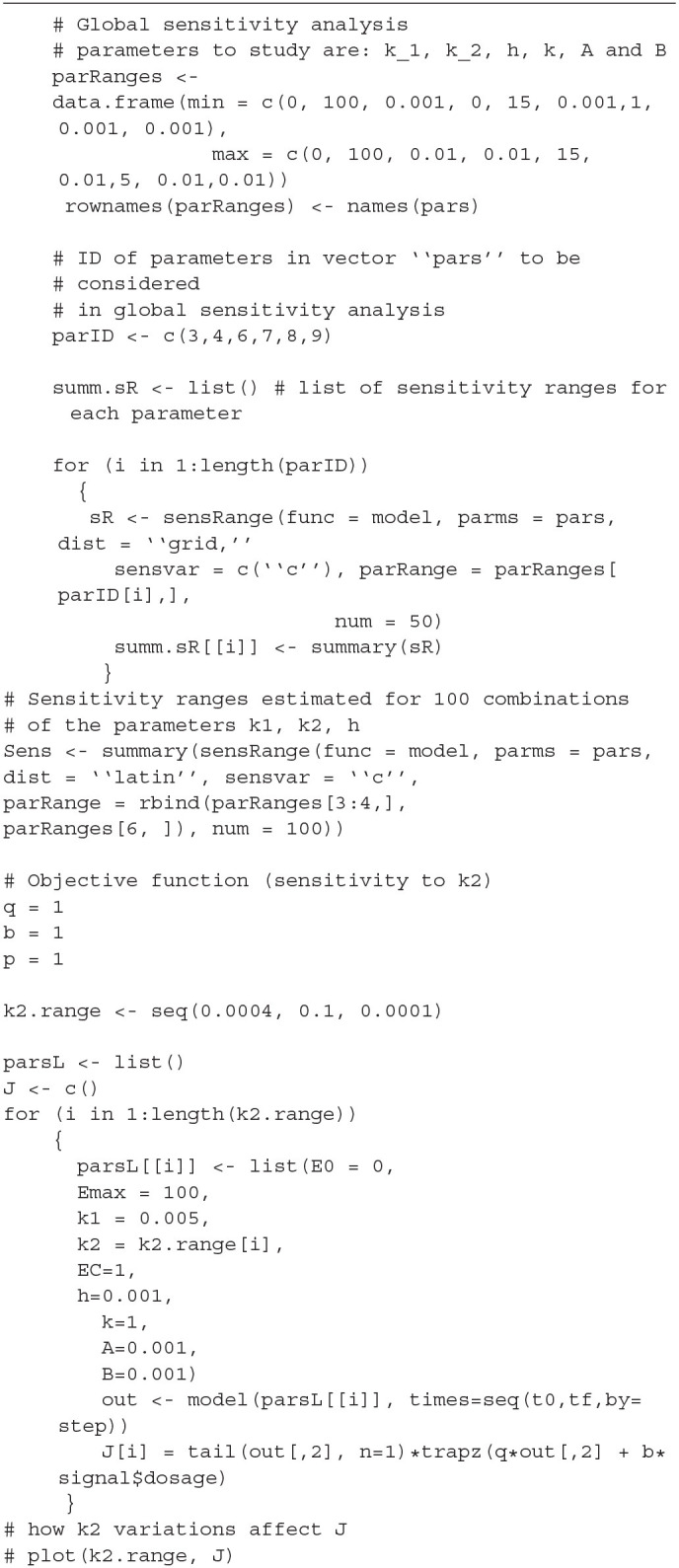

**Figure 2:**
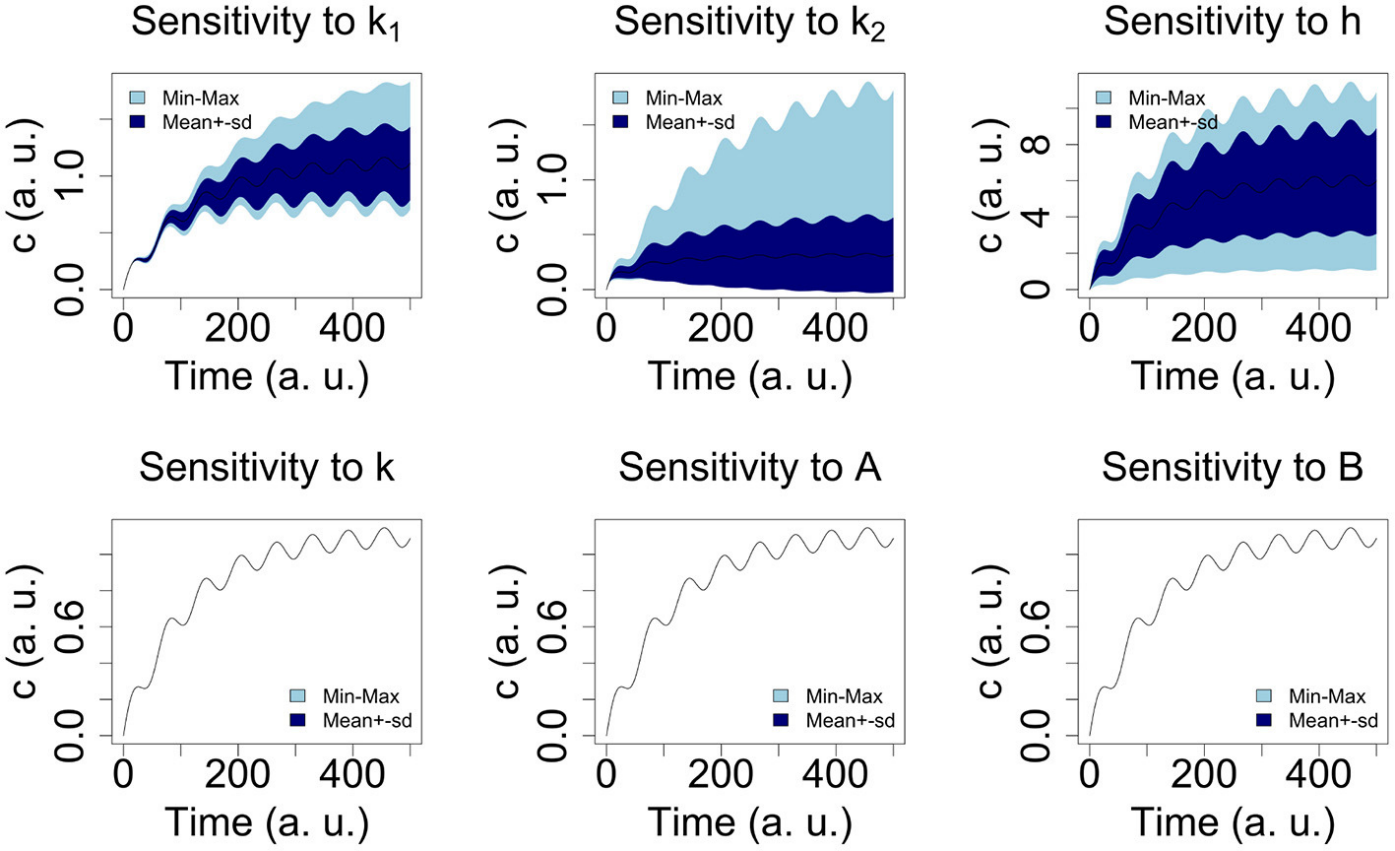
Sensitivity range of the concentration curve to the parameters listed in Table 3 (see text for R-code).

**Figure 3:**
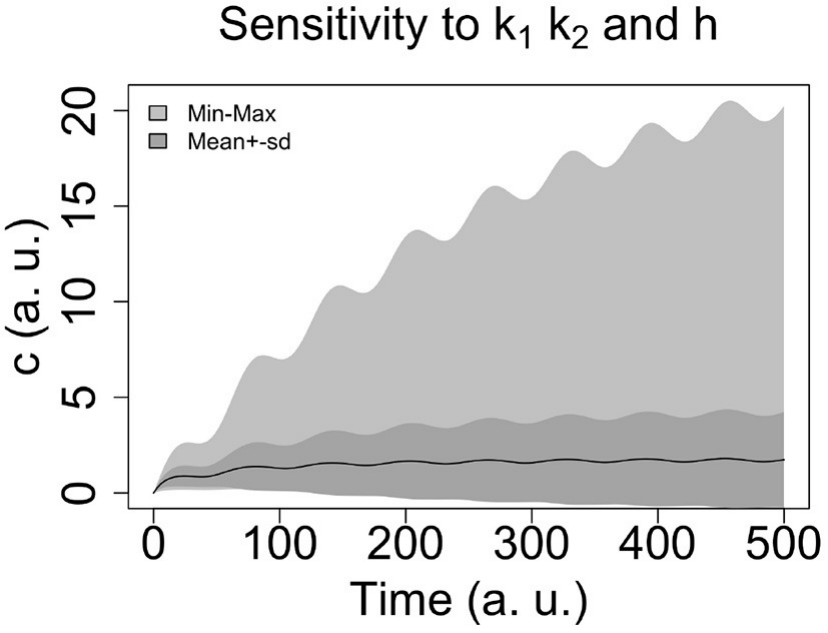
Sensitivity ranges of the concentration *c*(*t*) for 100 combination of parameters *k*_1_, *k*_2_ and *h*. We select the Latin hypercube sampling algorithm (see the R code in Table 2).

For the parameters *A, B, k*_1_, *k*_2_, and *k* we considered ranges of values (see Table 3) and carried out global sensitivity analysis of the model using the functions of the R library FME (Soetaert and Petzoldt, 2010). Figure 2 shows the sensitivity ranges of the concentration curve *c*(*t*) to the parameters listed in Table 3. The analysis shows that the model is sensitive to *k*_1_, *k*_2_, and *h*, and not sensitive to *k*, *A* and *B*. In Figure 3 sensitivity ranges of the concentration *c*(*t*) are reported for the combination of parameters *k*_1_, *k*_2_ and *h*.

**Table 3:** Parameters' ranges used for the global sensitivity analysis of the model defined by Equations (15) and (16).

**Parameter**	**Min**.	**Max**
*k*_1_	0.001	0.01
*k*_2_	0.000	0.01
*h*	0.001	0.01
*k*	1.000	5.00
*A*	0.001	0.01
*B*	0.001	0.01

These ranges have been obtained with the procedure reported in Hearne (1985), where we selected the latin hypercube sampling algorithm (see the R code in Table 2).

**Figure 4:**
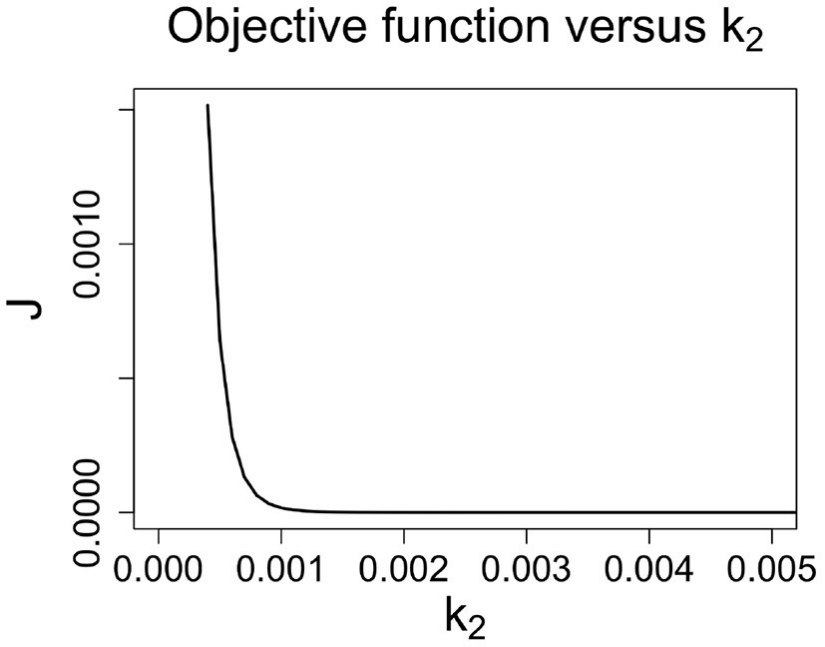
The value of objective function *j* rapidly decreases as *k*_2_ increases.

We focus our analysis on *k*_2_ (similar conclusions can be drawn for the parameter *h* since it is as *k*_2_ a multiplicative factor of *u*(*t*)). The higher the positive *k*_2_ value, the sooner the curve *c*(*t*) reaches the saturation point and the lower the concentration saturation value is in Figure 4, we see that how *k*_2_ affects the value of *J*: a small increment of *k*_2_ induces a steep decrement of *J*.

**Figure 5:**
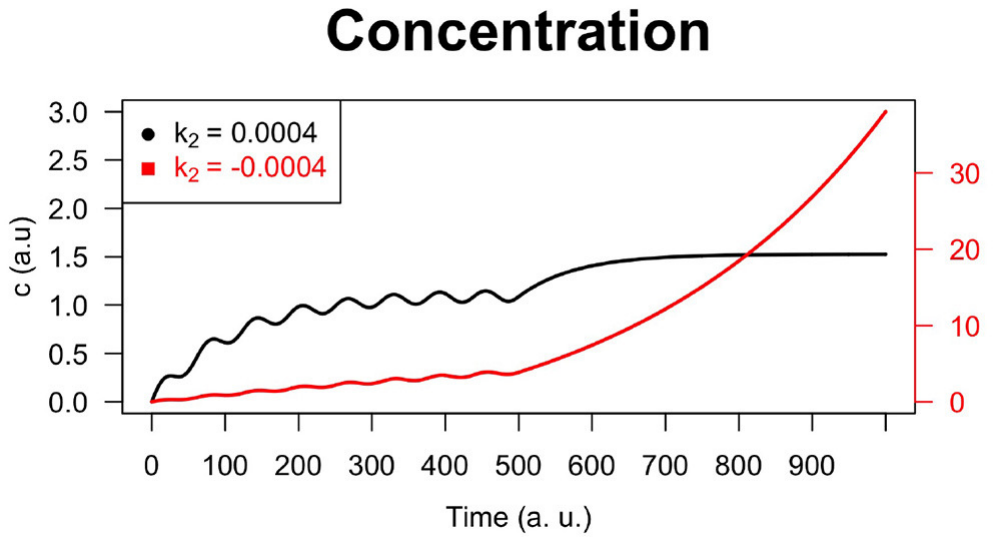
Numerical simulation of the system defined by Equations (15) and (16) with *E*_0_ = 0, *E*_max_ = 100, *k*_1_ = 0.005, *k*_2_ = −0.0004, *EC*_50_ = 15, *h* = 0.001, *k* = 1, *A* = 0.001, *B* = 0.001. A negative value of *k*_2_ causes the concentration curve to assume an increasing monotone trend, while a positive value of *k*_2_ causes the curve to reach a plateau after an initial oscillatory behavior.

An interesting case arises for negative values of *k*_2_. For example, if we consider *k*_2_ = −0.0004 (instead of *k*_2_ = +0.0004), the curve of *c*(*t*) looks like in Figure 5: after a time interval in which it shows slight fluctuations corresponding to the fluctuations of *u*(*t*), its growth accelerates and the fluctuations become almost nil. In case of negative values of *k*_2_ the control and the optimal control are not guaranteed. In this regard, Ledzewicz et al. (2013) comment saying for the bilinear model the sign *k*_2_ matters and for *k*_2_ > 0 singular controls satisfy the necessary conditions for optimality. There is an intuitive explanation for this: once the drug's concentration is built up, in this case the injection of smaller time-varying doses can be used to maintain a high effectiveness of the drug which by itself slowly decays.

We finally note that the scarce influence of parameters *A* and *B* on the model reveals the scarce influence of the compartment of the model in which the drug-cell interactions take place. This fact is a limitation of these models, since the processes through which the drug performs its functions are different in different phases of the cell cycle.

## 5. Potential Future Developments in the Field

Considering the model described in the previous section, it may look like we always know the explicit formulas for the control(s), but, unfortunately, this is not the case in most of the applications of control theory to the experimental sciences and especially to biological sciences. Usually, a guess of the analytical form of *u*(*t*) comes from the first experimental regime designed for drug administration scheduling. However, the example shown in the previous section shows that the knowledge of the mathematical form for *u*(*t*) is not enough if an accurate knowledge of its parameters is missing. We indeed observed that the controllability and also the optimality of the control strongly depend on the parameters of *u*(*t*) (*k*_2_ and *h* are two multiplicative factors of *u*(*t*)).

As Moore notes in Moore (2018), a frequent assumption in pharmacokinetic/pharmacodynamic modeling is that the structure of the equations is the same in animals and humans, and only the parameter values differ. In the case in which parameter values in a model are obtained by fitting the model to animal preclinical data. Allometric scaling is used to predict corresponding parameter values for a human population (Knibbe et al., 2005; Mahmood, 2009; Huang and Riviere, 2014). In spite of the fact that allometric scaling is a simple potential tool and rational option for the estimate of pharmacokinetic parameters in species for which there are no data available, is has no the same predictive power of the use of human data samples. if data from human cohorts are available, the best way to obtain the parameter values is by directly fitting the model to human clinical data. In addition to tying models closely to data, evaluation of a predicted optimal regimen with experimental data is very essential. Study outcomes from regimens that are predicted to be optimal should be compared with those from standard regimens (Moore, 2018). Such evaluation tests whether assumptions in the model, the objective functional, and the parameter estimates, are plausible and lead to desired outcomes (i.e., the model is optimally controllable by *u*(*t*)).

The experimental determination of a possible model for *u*(*t*) and the estimation of its parameters are not the last steps to be taken before applying the control theory to optimize or discard the proposed regime for *u*(*t*). Performing sensitivity analysis on the model is an equally important step that we suggest to perform before any attempt to fit the model to the available data. As we have seen in the simple example of the previous section, sensitivity analyses is crucial to identify parameters that the outcome is largely insensitive. The parameters that do not affect the model can be fixed, while the other can be estimated by fitting the model to data or from the literature. Finally, however, the presence of parameters to whose variations the model is insensitive deserve out attention, and has not to be welcome as the possibility to reduce the dimensions of the free parameter space. It could indicate the inadequacy of the model used to describe the kinetics and dynamics of the drug under investigation. This is the case for example of similar models to the one presented in this study, which in fact is representative of a populated class of similar models. The specificity of the kinetics and dynamics at the cell cycle phase that the chemotherapist demonstrates *in vivo* is not adequately described by these models. In such models, the controllability analysis does not depend on the actual values of these parameters, but it is the type of pharmacokinetics model which determines the class of optimal controls, as noted also by Ledzewicz et al. (2013). In this case the issue about the proper choice of pharmacokinetics in the cancer chemotherapy problems arises and become an essential item in the modeling of cancer chemotherapy. In fact, it must always be remembered that when we talk about controllability we always refer to the controllability of a *model*. If the model turns out to be very close to reality then, the terms *model* and *system* can be used interchangeably and it can be said whether the *system* (and not only the *model*) is controllable or not.

## 6. Conclusions

The techniques and examples in this article are intended to present to practitioners and modelers of pharmacokinetics/pharmacodynamics the basic concepts of the optimal control theory and its application to optimize drug regimens.

Considering a simple but widely used example we show that the prediction about optimal controllability depends on the formal characteristics of the model equation and the knowledge or the possibility to infer their parameters. Controllability is primarily a property of the model, which becomes a property of the system if the model is accurately realistic. By integrating clinical data with data concerning the molecular processes of drug metabolism, mathematical models that are able to accurately capture the drug kinetics and dynamics as well as the dynamics of the disease states, there are advantageous opportunities to take advantage of the application of optimal control theory. The final goal is to plan therapeutic regimens for preclinical or clinical use to predict optimal therapeutic regimens.

Until a few years ago the clinical application of mathematical models of tumor growth were limited because they require input data difficult to obtain with sufficient spatial resolution in patients even at a single time point—for example, extent of vascularization, immune infiltrate, ratio of tumor-to-normal cells, or extracellular matrix status (Yankeelov et al., 2013). Nowadays, however, the availability of data from advanced quantitative tumor imaging methods rekindles the interest in the development of mathematical models and paves the way to an effective application of control theory to predict optimal therapy delivery parameters and ultimately to control the response to treatment and the time to progression. Indeed, the number of publications on mathematical modeling of cancer is growing at an exponential rate (Brady and Enderling, 2019), and for this reason it is urgent to consider the application of control theory to the analysis of the available models to evaluate their effectiveness as tools to aid in the programming of an optimal dosage and drug administration scheduling.

In this direction, there is also another particularly important aspect that control theory must incorporate, namely the development of the chemo-resistance. In a recent study concerning the abiraterone treatment for metastatic castrate-resistant prostate cancer, Gatenby et al. (Zhang et al., 2017a) noted that with standard dosing, evolution of resistance with treatment failure (testified by radiographic progression) occurs at a median of about 16.5 months.

The authors observed that the conventional treatment strategy, which administers cytotoxic drugs at maximum tolerated dose until progression, can be evolutionarily unwise since it strongly selects for resistant phenotypes and eliminates potential competitors. These Darwinian dynamics can lead to rapid proliferation of resistant populations. On the basis of this idea, the authors developed an evolutionary game theory model of Lotka–Volterra equations with three competing cancer “species”: androgen dependent, androgen producing, and androgen independent. As expected, the model simulations with standard abiraterone dosing demonstrated strong selection for androgen-independent cells and rapid treatment failure. Gatenby et al. then hypothesized that the time to progression could be increased by integrating evolutionary dynamics into therapy scheduling models. In order to implement this integration, they framed the population dynamics before and during abiraterone therapy using a game theoretic model built on evolutionary first principles with parameter estimates inferred from clinical data. The results of Gatenby et al. model (Zhang et al., 2017a) demonstrated how a therapy treatment strategy synchronized with intra-tumoral evolutionary dynamics may prolong time to progression and significantly decrease total drug dose. The evolution-based strategy suggested by the model was then applied in pilot clinical trials.

Cancer is a complex multiscale process (Zhang et al., 2008; Deisboeck et al., 2011), whose modeling requires the integration of different type of data (Deisboeck et al., 2011). this raises the problem of developing control theory approaches to multiscale systems. Building a model for a multiscale system is generally challenging. In particular, a multiscale model of tumoral growth in response to a therapy, can include (i) equations spanning a wide range of time and length scales, (ii) stochasticity inherent to the intracellular and intercellular biochemical interactions, and, consequently, non-continuum and dynamically coupled continuum-non-continuum processes description. To all this is added a comprehensive knowledge of the physicochemical mechanisms as well as the values of thermodynamic and kinetic parameters of the biochemical reactions involving the drug and its target molecules. Each of these points poses a challenge to the control theory applied to tumoral growth models. Overcoming each of these challenges in a short time may be possible if in the near future we will be able to develop a control theory suitable for the analysis of controllability of multiscale hybrid stochastic systems.

From the theoretical point of view, in the last decade works have been published that expose the mathematical foundations of control theory or that do discuss the perspectives of its application to the case of multiscale systems (see for example (Christofides and Armaou, 2006; Christofides et al., 2009; Hartmann et al., 2014; Mei et al., 2016), and to stochastic systems (see for example (Braatz et al., 2006; Nisio, 2015; Zhang et al., 2017b). What is currently lacking and which could prove to be of great use for the design of dosages and administration regimens of an anticancer drug is the construction of a control theory of control for hybrid stochastic multiscale systems. The modeling and control of stochasticity could also provide further tools for controlling the evolution of drug resistance (as an extension of evolutionary dynamics models) and implementing appropriate countermeasures.

## Author Contributions

PL has carried out the bibliographic research necessary to review the state of the art of the application of control theories to pharmacokinetic/pharmacodynamic models and is the author of the design and R codes that implement the analysis of these models.

## Conflict of Interest

The author declares that the research was conducted in the absence of any commercial or financial relationships that could be construed as a potential conflict of interest.
